# CXCL13 is androgen-responsive and involved in androgen induced prostate cancer cell migration and invasion

**DOI:** 10.18632/oncotarget.18387

**Published:** 2017-06-07

**Authors:** Long Fan, Qingyi Zhu, Li Liu, Cuicui Zhu, Haojie Huang, Shan Lu, Ping Liu

**Affiliations:** ^1^ Jiangsu Province Key Laboratory for Molecular and Medicine Biotechnology, Life Science College, Nanjing Normal University, Nanjing, Jiangsu, China; ^2^ Department of Urology, Jiangsu Province Hospital of TCM, Nanjing, Jiangsu, China; ^3^ Laboratory of Molecular Biology, Jiangsu Province Hospital of TCM, Nanjing, Jiangsu, China; ^4^ Mayo Clinic Cancer Center, Mayo Clinic College of Medicine, Rochester, MN, USA

**Keywords:** androgen, androgen receptor, chemokine, CXCL13, prostate cancer

## Abstract

Androgen receptor (AR) is a key transcription factor playing a critical role in prostate cancer (PCa) initiation and progression. However, the molecular mechanisms of AR action in prostate cancer are not very clear. CXCL13, known as B cell attracting chemokine1 (BCA-1), is a member of CXC chemokine family and relevant to cancer metastasis. This study shows that CXCL13 is an androgen-responsive gene and involved in AR-induced PCa cell migration and invasion. In clinical specimens, expression of CXCL13 in PCa tissues is markedly higher than that in adjacent normal tissues. In cultures, expression of CXCL13 is up-regulated by androgen-AR axis at both mRNA and protein levels. Furthermore, Chip-Seq assay identifies canonical androgen responsive elements (ARE) at CXCL13 enhancer and dual-luciferase reporter assays reveals that the ARE is highly responsive to androgen while mutations of the ARE abolish the reporter activity. Additional chromatin immunoprecipitation (ChIP) assays also identify that the ARE presents androgen responsiveness. In addition, CXCL13 promotes G2/M phase transition by increasing Cyclin B1 levels in PCa cells. Functional studies demonstrate that reducing endogenous CXCL13 expression in LNCaP cells largely weakens androgen-AR axis induced cell migration and invasion. Taken together, our study implicates for the first time that *CXCL13* is an AR target gene and involved in AR-mediated cell migration and invasion in primary PCa.

## INTRODUCTION

Prostate cancer (PCa) is the most commonly diagnosed malignancy and the predominant cause of death from cancer among men in the western countries [1]. Androgen and androgen receptor (AR) have always been a leading driving force for PCa and played significant roles in both the beginning and progression of PCa [2–4]. Metastasis is always a primary cause of mortality in prostate cancer even though there have been continual improving techniques and methods in diagnosis and treatment [5]. Bone metastasis is a common conundrum for majority of cancers and occurs in up to 70% patients with advanced PCa [6]. Although it is well known that androgen and AR promote PCa cell metastasis, the molecular mechanisms of androgen-mediated cell metastasis remain poorly understood.

Chemokines (chemotactic cytokines) are a family of low molecular weight cytokines, that have a crucial regulation effect in diverse immunoinflammatory responses through their interaction with specific receptors and activation of specific leukocytes [7, 8]. Based on the pattern of their N-terminal cysteines, more than 40 different human chemokines have been divided into four conserved groups: ‘CXC’ chemokines, ‘CC’ chemokines, ‘XC’ chemokines and ‘C3XC’ chemokines [8, 9]. Recently, increasing evidence suggests that the complex chemokine network plays a key role in cancer cell survival, growth, migration, invasion, proliferation, and apoptosis [10–14]. For example, CXCL12 is approved to be involved in regulating bone marrow metastases in breast cancers and small-cell lung cancer [15, 16], and stimulate cell migration and invasion in ovarian cancer [17]. CXCL13 is also showing in regulating lymphocyte migration and promoting inflammation and acts as a new target for the detection and treatment of lymphoma [18–21]. In addition, CXCL13 is over-expressed in breast cancer and colon cancer patients and tightly related with the poor prognosis and overall survival of patients [22–24]. In PCa, serum CXCL13 levels is positively correlated with prostate specific antigen and prostatic disease; and PCa cell lines selectively express certain matrix metalloproteinases (MMPs) in response to aberrant expression of CXCL13, suggesting that migration and invasion of PCa cells is via degrading extracellular matrix (ECM) components [25, 26]. However, the effect of CXCL13 on AR-induced PCa cell migration and invasion is still unknown.

In this study, we reported for the first time that expression of CXCL13 was up-regulated by androgen/AR axis in PCa cells and clarified *CXCL13* as a novel downstream target gene of AR. By using ChIP-Seq, ChIP assay and dual-luciferase reporter assays, we identified that the functional androgen responsive elements (ARE) were contained in human *CXCL13* enhancer. Data of over-expression and knock-down of CXCL13 demonstrated that CXCL13 impaired androgen/AR-induced up-regulation of PCa cell migration and invasion. Accordingly, we conclusively illuminated that, as a potential target gene of AR, CXCL13 is involved in the process of androgen/AR axis-enhanced PCa progress. It might be a valuable clue in clinic for digging out new drugs and treatment methods on patients with prostate cancer.

## RESULTS

### Expression of CXCL13 in PCa tissues and cells

**Figure 1:**
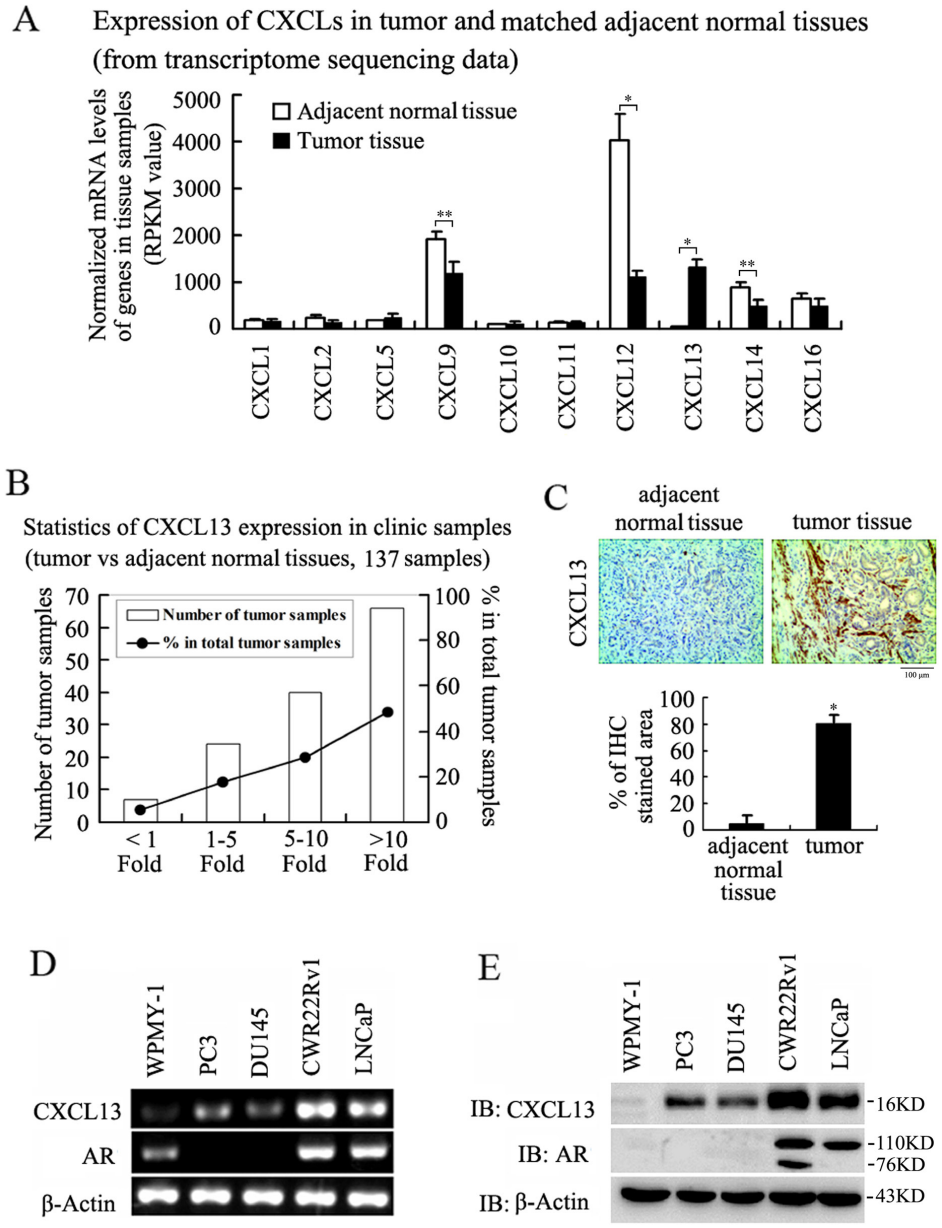
Expression of CXCL13 in PCa tissues and cell lines **(A)** Expression of some CXC chemokines family members in tumor and matched adjacent normal tissues from transcriptome sequencing data Basic Analysis. *p < 0.01, **p < 0.05. **(B)** Representative results of qRT-PCR analysis of the increased degree of CXCL13 mRNA expression in 137 PCa tissues matched with the adjacent normal tissues. **(C)** Immunohistochemistry detection of CXCL13 expression in PCa tissues and the adjacent normal tissues. The brown color means CXCL13 positive expression. *p < 0.05. **(D** and **E)** The expression of CXCL13 at mRNA (D) and protein (E) levels in normal prostate epithelial cell line WPMY-1 and four PCa cell lines: androgen-dependent LNCaP and CWR22Rv1 cell lines, androgen-independent PC3 and DU145 cell lines.

According to the results of Basic Analysis from transcriptome sequencing data (done by our lab), some detectable CXC chemokine family members were distinctly different expression between primary PCa tissues and matched adjacent normal tissues. Among them, CXCL9, CXCL12, CXCL14, CXCL16 had decreased expression, while CXCL13 had increased expression in the PCa tissues compared with the adjacent normal tissues (Figure 1A). In order to confirm the high expression of CXCL13 in primary PCa tissues, we performed qRT-PCR analysis in 137 clinical samples (Gleason score were 7-10), 7 (5.11%) showed less than 1-fold increased, 24 (17.52%) showed 1∼5-fold increased, 40 (29.20%) showed more than 5∼10-fold increased and 66 (48.18%) showed more than 10-fold increased (Figure 1B). Notably, the protein levels of CXCL13 were also markedly increased in PCa tissues compared with matched adjacent normal tissues (Figure 1C). *In vitro*, we found that levels of CXCL13 were abundant in androgen-responsive LNCaP and CWR22Rv1 cell lines compared to androgen-unresponsive DU145 and PC3 cell lines at both mRNA and protein levels, and the expression of CXCL13 in normal prostate epithelial cell line WPMY-1 was the lowest (Figure 1D and 1E). Thus, the results indicated that the high expression of CXCL13 was significantly correlated with prostate tumor in clinic, and the expression of CXCL13 might be up-regulated by androgen/AR axis.

### Expression of CXCL13 was correlated to androgen

**Figure 2:**
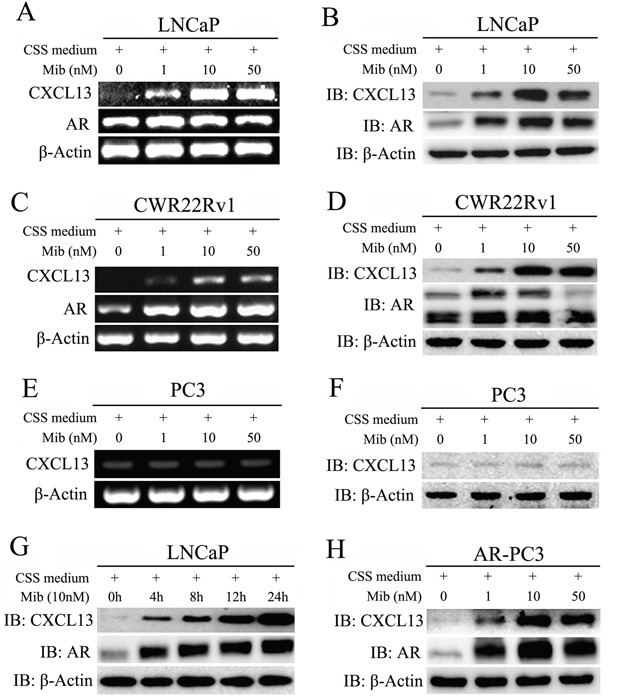
Androgen stimulation enhanced expression of CXCL13 in AR expressed PCa cells **(A, B, C, D, E** and **F)** After stimulation with different dose of Mib for 24 h, LNCaP, CWR22Rv1 and PC3 cells were harvested and treated for RT-PCR analysis (A, C and E) and Western-blot assay (B, D and F) to examine the expression of *CXCL13*, *AR* and *β-actin* (as internal control) genes. **(G)** LNCaP cells were stimulated with 10 nM mibolerone (Mib) for different times and then harvested for western-blot assays to detect the protein levels of CXCL13, AR, and β-actin. **(H)** Protein levels of CXCL13, AR, and β-actin (as internal control) assayed by western-blot in AR transient transfected PC3 cells with the stimulation of different dose of Mib as indicated.

To explore whether that expression of CXCL13 is up-regulated by androgen, AR-positive human PCa cell lines LNCaP and CWR22Rv1 were respectively hormone-stripped for 3 days (cells cultured in CSS medium), and then treated with different doses of mibolerone (Mib), a synthetic potent anabolic androgen which is both high affinity and selectivity for AR. As shown in Figure 2A-2D, Mib treatment increased both mRNA and protein levels of CXCL13 in a dose-dependent manner in two cell lines; and Mib at 10 nM had the most effect on the up-regulation of CXCL13 protein levels by comparing with other concentrations. Moreover, treatment of 10 nM Mib led to a time-dependent increase of CXCL13 protein levels in LNCaP cell after hormone-stripped for 3 days (Figure 2G). However, induction of CXCL13 by Mib treatment was not seen in AR-negative PC3 cells (Figure 2E and 2F). After transfection of AR plasmids in PC3 cells (or in AR-stable PC3 cells, data not shown), the expression of CXCL13 was Mib responsive again as showed in LNCaP and CWR22Rv1 cells (Figure 2H). Furthermore, in the LNCaP and CWR22Rv1 cells cultured in hormone-stripped medium without Mib treatment, the expression of CXCL13 was the lowest one due to the inactivated AR (Figure 2A-2D, 2G, and 2H). Accordingly, the expression of CXCL13 could be induced by androgen in various human PCa cell lines, and might be directly mediated by AR.

### CXCL13 was a novel downstream target gene of and regulated by androgen receptor

**Figure 3:**
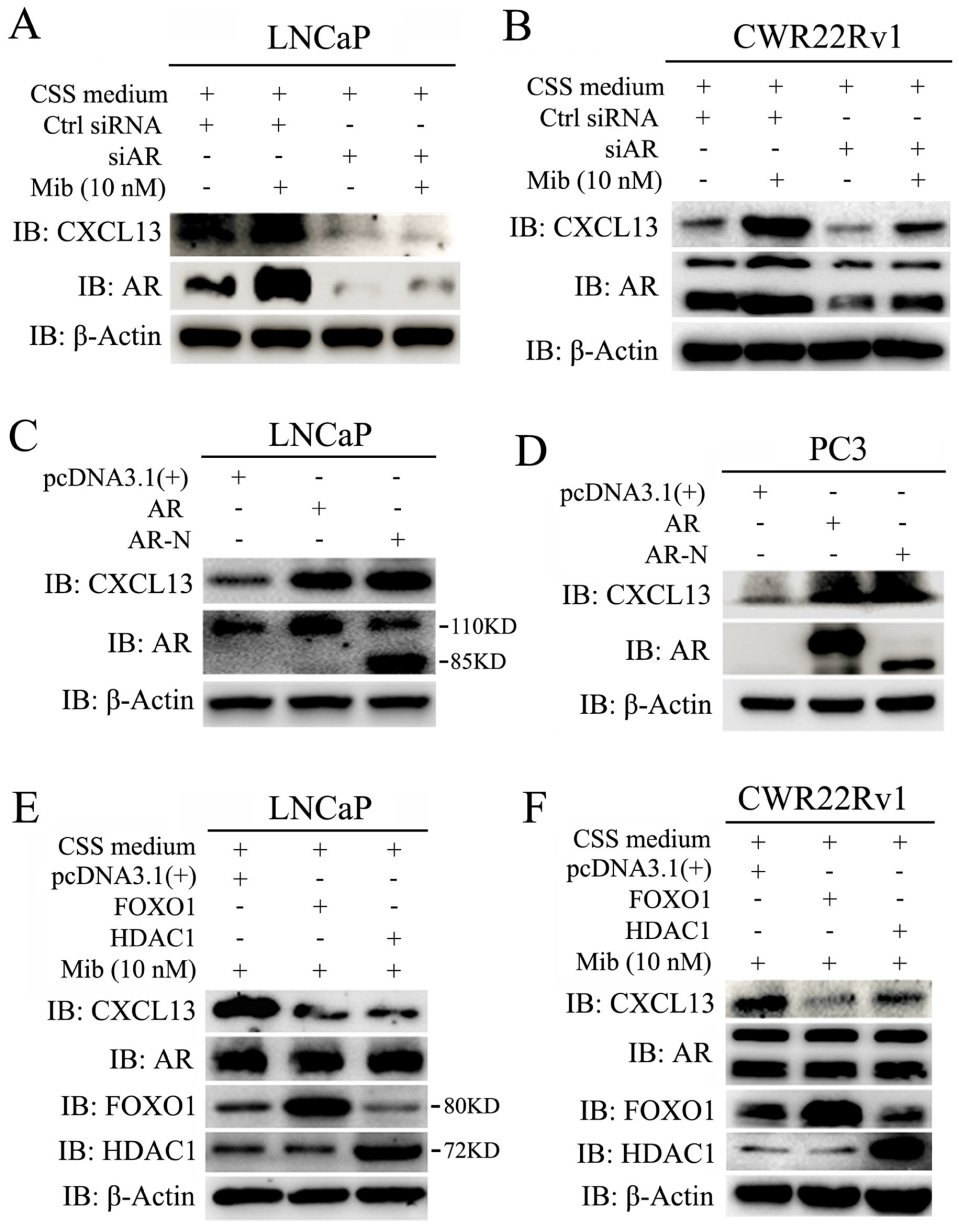
CXCL13 was a novel downstream target gene of AR **(A** and **B)** LNCaP (A) and CWR22Rv1 (B) cells were transfected with siAR (AR siRNA)/control siRNA (Ctrl siRNA) and then treated with or without 10 nM Mib. Cells were harvested for western-blot assay to examine the protein levels of CXCL13, AR and β-actin. Knockdown of AR induced down-regulation of CXCL13 in both cell lines. **(C** and **D)** LNCaP cells (C) and PC3 cells (D) were transfected with AR, AR-N (a constitutively active isoform) and control vector pcDNA3.1(+). Cells were harvested for western-blot assay to examine the protein levels of CXCL13, AR and β-actin. Over-expression of AR and AR-N induced up-regulation of CXCL13 in both cell lines. **(E** and **F)** LNCaP cells (E) and CWR22Rv1 cells (F) were separately transfected with FOXO1, HDAC1 and pcDNA3.1(+) and then stimulated with 10 nM Mib as indicated. Cells harvested for western-blot assay to examine the protein levels of CXCL13, AR, FOXO1, HDAC1 and β-actin. Over-expression of FOXO1 and HDAC1 induced down-regulation of CXCL13, but no effect on AR. These experiments were repeated at least three times.

To confirm the induction of CXCL13 by Mib is mediated by AR, we carried out knock-down of AR by transfecting small interfering RNA (siRNA) in LNCaP and CWR22Rv1 cells, respectively. Immediately after transfection, cells were cultured in 10% CSS medium for 48 hr and then treated with or without 10 nM Mib for another 24 hr. As shown in Figure 3A and 3B, knock-down of endogenous AR not only decreased basal level of CXCL13 in Mib-unstimulated group, but almost completely abrogated Mib-induced increase of CXCL13 expression. In addition, we transfected AR and AR-N (a constitutively active isoform) in LNCaP and PC3 cells, after 48 hr, cells were harvested for western-blot assay. Results showed that CXCL13 were apparently up-regulated by transient over-expressed AR and AR-N (Figure 3C and 3D). Moreover, we transfected LNCaP and CWR22Rv1 cells with expression plasmids of FOXO1 and HDAC1, which both of them have been reported to repress AR activity [27–29]. In order to effectively testify the effect of activity-repressed AR on the expression of CXCL13, transfected cells were cultured in 10% CSS medium for 48 hr and then treated with 10 nM Mib for another 24 hr. As shown in Figure 3E and 3F, CXCL13 protein levels were dramatically decreased by transient over-expressed FOXO1 and HDAC1, respectively; whereas the protein levels of AR were not affected by FOXO1 and HDAC1. Thus, we concluded that *CXCL13* was the downstream target of androgen receptor and the expression of *CXCL13* was regulated by androgen/androgen receptor (AR) axis.

### AR bound the enhancer of *CXCL13* gene to up-regulate its expression

**Figure 4:**
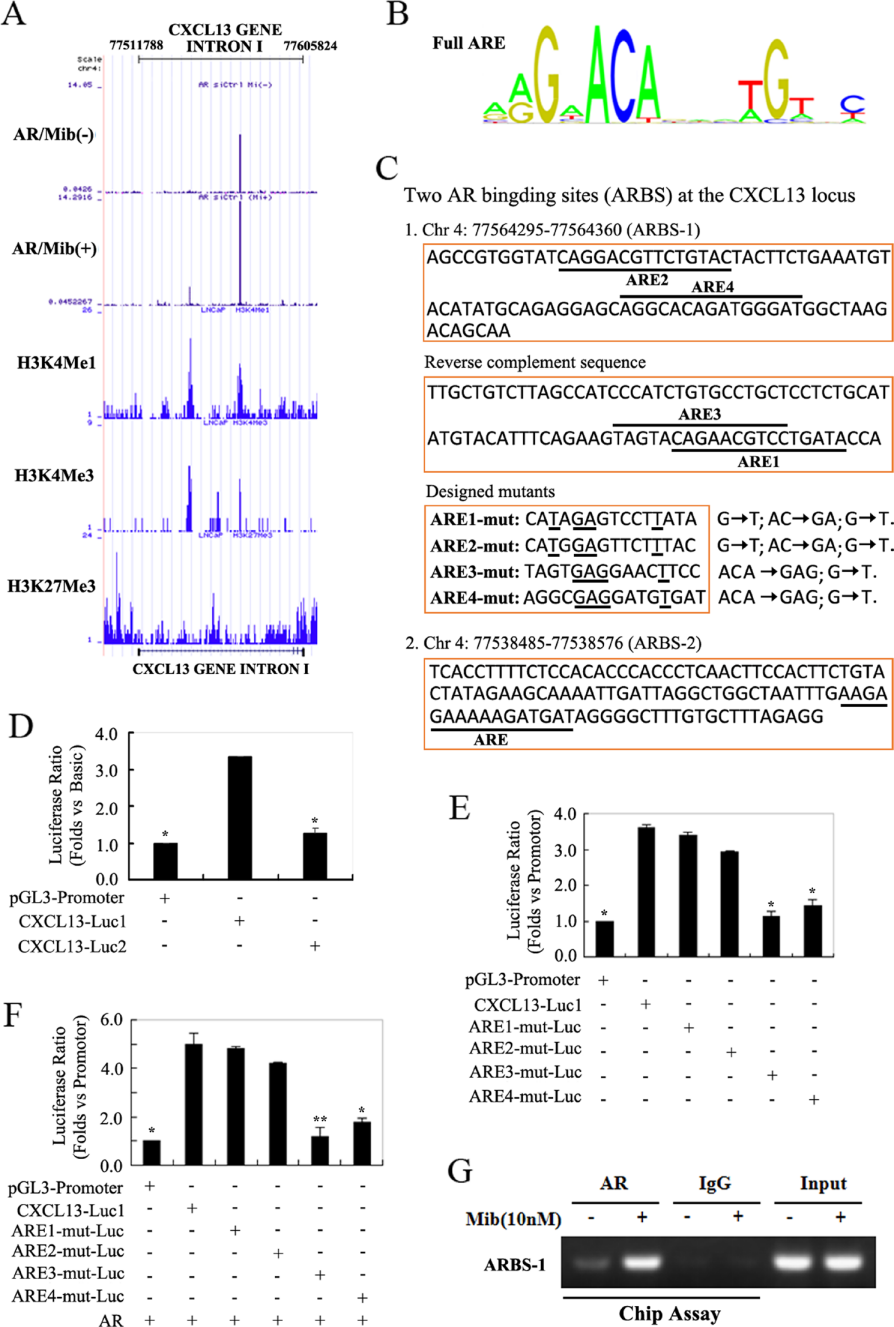
AR regulated the expression of CXCL13 by targeting its enhancer sequences **(A)** AR Chip-seq data in LNCaP cells showed two combined peaks within CXCL13 intron I (enhancer region of *CXCL13* gene). **(B)** Canonical full-androgen response element (ARE) sequences. **(C)** The AR-binding sites in CXCL13 intron I predicted by website JASPAR, including ARBS-1 (*upper*, four putative AREs with underline; *down*, designed mutants of key residues in these AREs) and ARBS-2 (contains one putative ARE with underline). **(D)** LNCaP cells transfected with reporter plasmids and renilla luciferase reporter gene were subjected to measure luciferase activity. The measured luciferase values were all normalized with renilla. The luciferase ratios were obtained by considering pGL3-promoter value as 1. **(E** and **F)** Cells (E, LNCaP cells; F, PC3 cells) transfected with reporter plasmids, renilla luciferase reporter gene and/or AR were subjected to measure luciferase activity. The normalized luciferase values and luciferase ratios were also obtained as the description above. **(G)** It's the Chip-assay data. LNCaP cells cultured in 10% CSS medium and treated with 10 nM Mib as indicated in figure were harvested for Chip-assay. DNA immunoprecipitated with AR antibody and IgG (as a nonspecific control) were used as templates for PCR using primers that amplified the AR binding site 1 (ARBS-1) within CXCL13 intron I. The same PCR products from the DNA before immunoprecipitation were loaded as the input. Luciferase experiments were repeated three separately times. * *p* < 0.01, ** *p* < 0.05.

AR is known to be the primarily function as a transcription factor by binding the enhancers of its target genes [4]. To determine the molecular basis for the induction of CXCL13 by androgen/AR axis, we went on to identify the androgen-responsive sequences in CXCL13 gene area. By analyzing AR ChIP-seq data in LNCaP cells (done by Haojie Huang's Lab, Mayo Clinic Cancer Center, Rochester, MN, USA), we found that there are two obvious AR binding peaks located within CXCL13 intron I. Corresponding to these two peaks of CHIP-seq results, there were two AR-binding sites (called as ARBS-1 and ARBS-2) in CXCL13 intron I; and the abundance of AR binding at the two sites were markedly increased by following Mib treatment (Figure 4A). By further using transcription factor binding sequence prediction website JASPAR, four canonical androgen responsive elements (ARE) sequences (Figure 4B) were separately identified in ARBS-1 and ARBS-2 within CXCL13 intron I (Figure 4C). To investigate the effects of these two ARBS in androgen responsiveness, we constructed two luciferase reporter plasmids (named CXCL13-Luc1 and CXCL13-Luc2) separately containing the two ARBS sequences and then transfected the plasmids into LNCaP cells for luciferase assay. The results of dual-luciferase assay in LNCaP cells showed that ARBS-1 (CXCL13-Luc1) was found to have approximately 3.3-folds increased in luciferase activity by comparing with pGL3-promoter (empty vector), while no obvious luciferase activity changes were observed in ARBS-2 (CXCL13-Luc2; Figure 4D). Furthermore, we generated luciferase reporter plasmids containing key nucleotides mutations within the four ARE sequences of ARBS-1 (called as ARE1-mut, ARE2-mut, ARE3-mut and ARE4-mut), and the mutation strategy was shown in Figure 4C. Upon transfection of reporter plasmids into LNCaP cells, the luciferase activities of ARE3-mut and ARE4-mut were obviously decreased by comparing with the luciferase activity of ARBS-1 (CXCL13-Luc1). Moreover, mutant ARE3-mut (mutated at the ARE3 sequence) almost completely abrogated the increased luciferase activity of ARBS-1 (CXCL13-Luc1; Figure 4E). Similar results were also obtained in PC3 cell when co-transfected with AR expression plasmid (Figure 4F). All the data indicated that ARE3 and ARE4 were the potential cardinal androgen responsive elements in CXCL13 gene.

To further verify the ARBS-1 of CXCL13 gene is the androgen responsive element (ARE), CHIP assays were carried out next. LNCaP cells were cultured in 10% CSS medium for 3 days and then treated with or without 10 nM Mib for 3 hr. The soluble chromatins pulled down by AR antibodies (IgG as the control) were used for CHIP assay. Shown as in Figure 4G, PCR analysis using specific primers designed to amplify the ARBS-1 region revealed a significant increment of AR binding in response to Mib stimulation, suggesting this region is the bona fide AR-binding region within CXCL13 enhancer.

### AR targeted genes, ETS-1, Snail and Cyclin B1, via CXCL13

**Figure 5:**
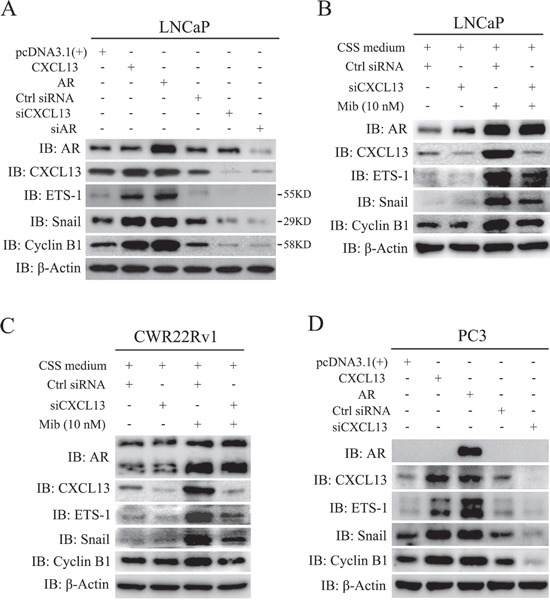
AR-induced genes, ETS-1, Snail and Cyclin B1, were mediated by CXCL13 **(A** and **D)** LNCaP and PC3 cells were transfected with CXCL13, AR, siCXCL13, siAR (just LNCaP cells), pcDNA3.1(+) and control siRNA (Ctrl siRNA) for 48 hr and then harvested and lysed in lysis buffer. The protein levels of ETS-1, Snail, Cyclin B1, CXCL13, AR and β-actin were checked using western-blot assay and showed in figures. **(B** and **C)** LNCaP cells (B) and CWR22Rv1 cells (C) were transfected with siCXCL13 and control siRNA (Ctrl siRNA), and immediately switched to 10% CSS medium for 48 hr. After treating cells with or without 10 nM Mib for additional 24 hr, cells were harvested and lysed. The protein levels of ETS-1, Snail, Cyclin B1, CXCL13, AR and β-actin were checked by western-blot assay and the results were sowed in figures.

To explore the role of CXCL13 in AR mediated progression of PCa, we performed assays as below. As show in Figure 5A, LNCaP cells were transfected with expression plasmids of AR and CXCL13, siRNAs of siCXCL13 and siAR, respectively. After 48 hr, cells were harvested for immunoblotting assay. Results showed that transient over-expression of both AR and CXCL13 induced up-regulation of ETS-1, Snail and Cyclin B1 protein levels; whereas transfection of both siCXCL13 and siAR down-regulated the expression of ETS-1, Snail and Cyclin B1. Especially, knock-down of CXCL13 resulted in the lower protein levels of ETS-1, Snail and Cyclin B1 by comparing with that of cells transfected with pcDNA3.1(+) and control siRNA (Figure 5A). It meant that CXCL13 might involved in the up-regulation of some AR-targeted genes. To further identify that AR-induced ETS-1, Snail and Cyclin B1 up-regulation was mediated by CXCL13, LNCaP cells were next cultured in 10% CSS medium immediately after endogenous CXCL13 were knocked down by siCXCL13. After 48 hr, cells were treated with or without 10 nM Mib for another 24 hr. Immunoblotting results showed that Mib treatment induced the expression of ETS-1, Snail, and cyclin B1 apparently, while this induction was dramatically weakened by knocking down of endogenous CXCL13 (Figure 5B). Similar results were also obtained in CWR22Rv1 cells (Figure 5C). Additionally, in PC3 cells, transient over-expression of AR or CXCL13 enhanced the expression of ETS-1, Snail and Cyclin B1, while knock-down of endogenous CXCL13 also decreased the expression of ETS-1, Snail and Cyclin B1 (Figure 5D). Therefore, AR induced up-regulation of ETS-1, Snail, and Cyclin B1 was mainly mediated by CXCL13.

### CXCL13 involved in AR-induced cell migration and invasion in androgen-dependent PCa cells

**Figure 6:**
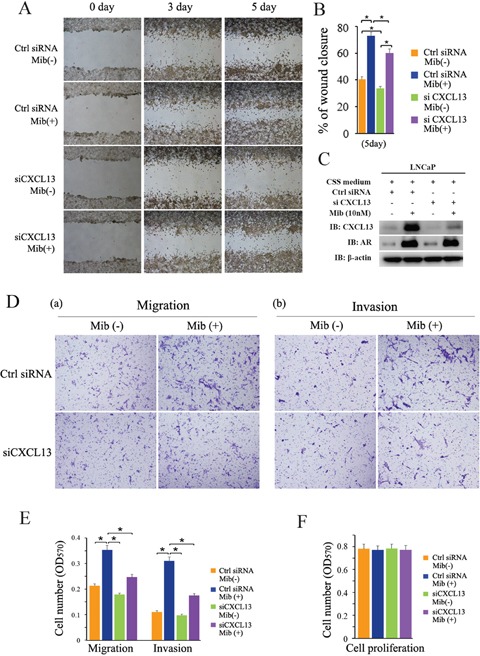
CXCL13 involved in androgen/AR-induced migration and invasion of PCa cell **(A)** LNCaP cells were transfected with siCXCL13 or control siRNA and cultured in 10% CSS medium. After 48 hr, cells were treated with or without 10 nM Mib, and the wound healing assay was performed. The area of the gaps of wound closure were determined by photography at 0 day, 3 days, and 5 days after Mib treatment and the results were showed here. **(B)** Percentages of wound closure in wound healing assays were quantified (the wound closure at 0 day as 100%). **(C)** The cells treated for wound healing assay were harvested for western-blot assay to validate the efficiency of CXCL13 knock-down in wound healing assay experiments. **(D)** LNCaP cells were transfected with siCXCL13 or control siRNA (Ctrl siRNA) and cultured in 10% CSS medium. After 48 hr, cells were re-suspended in both serum and phenol red free RPMI-1640 medium and treated with or without 10 nM Mib. And then transwell assays were carried out. The cells traversed the membrane were stained with 0.1% crystal violet and photographed. The data showed that CXCL13 promoted cell migration and also involved in AR-mediated PCa cell migration. **(E)** The quantification of migration and invasion assay in (D). **(F)** LNCaP cells were transfected with siCXCL13 or control siRNA (Ctrl siRNA) and cultured in 10% CSS medium for 48 hr. Cells were re-seeded in 96 plates treated with or without 10 nM for 24 hr. Cells were harvested for MTT assay. Error bars indicate s.d. among three individual experiments. *P<0.05.

Androgens have been reported to promote cell invasion and metastasis in androgen dependent PCa cells [30]. Previously studies revealed CXCL13 can regulate cancer cell migration and invasion [31, 32]. To determine whether CXCL13 is involved in AR-induced cellular migration and invasion in androgen dependent PCa cells, LNCaP cells were cultured and transfected as indicated in Figure 6 for performing wound healing assay and transwell migration/invasion assay. Results showed that knock-down of endogenous CXCL13 by siRNA distinctly weakened Mib-induced up-regulation of LNCaP cell migration (Figure 6A and 6D(a)). Quantitative analyses of cell migration (including wound healing assay and transwell assay) were performed and the quantified data are shown in Figure 6B and 6E. Knock-down of CXCL13 by siRNA and Mib-stimulated CXCL13 expression were confirmed by western-blot assay (Figure 6C). Additionally, knock-down of CXCL13 by siRNA also largely weakened Mib-induced up-regulation of LNCaP cell invasion through Matrigel matrix (Figure 6D(b)). Quantitative analyses of invasion cells were shown as Figure 6E. Notably, no significant effect on proliferation of LNCaP cells by knocking down CXCL13 with or without of 10 nM Mib treatment in 24 h (Figure 6F) indicated that cellular migration and invasion (by transwell assays) above were not caused by cellular proliferation. These results suggested that CXCL13 played an important role in AR-induced cellular migration and invasion in androgen dependent PCa cells.

### CXCL13-promoted cell migration and invasion were impaired by AR in androgen-independent PCa cells

**Figure 7:**
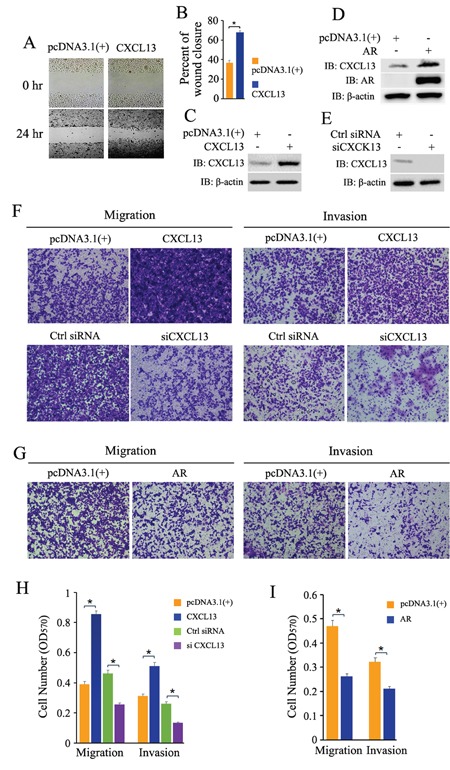
Effect of CXCL13 on cell migration and invasion in androgen-independent PCa cells with or without recovery expression of AR **(A)** PC3 cells transfected with CXCL13 and pcDNA3.1(+) were cultured in 1% FBS medium over night, and then the wound healing assay was carried out. The area of the gaps of wound closure were determined by photography at 0 hr and 24 hr. **(B)** Percentages of wound closure in wound healing assays were quantified (the wound closure at 0 hr as 100%). **(C, D** and **E)** The cells with the same treatment as in wound healing assays were harvested to validate the transfection efficiency of CXCL13, AR and siCXCL13 in the experiments (C and D, over-expressions of CXCL13 and AR in PC3 cells; E, knock-down of CXCL13 in PC3 cells). **(F** and **G)** PC3 cells were transfected with CXCL13, AR, pcDNA3.1(+), siCXCL13 or control siRNA as indicated in figures. 12 hr later, cells were switched to 0.05% FBS medium and cultured for another 12 hr, and then cells were re-suspended in RPMI-1640 with 0.05% FBS for cell migration and invasion test by transwell assay. The cells raversed the membrane were stained with 0.1% crystal violet and photographed. (**H** and **I**) The quantification of migration and invasion assay in (F) and (G). Error bars indicate s.d. among three individual experiments. *P<0.05.

PC3 cells were seeded and cultured overnight, and then transfected with plasmids and siRNAs as indicated in Figure 7. The wound healing assay and transwell migration/invasion assay were carried out. From our data, transient over-expression of CXCL13 apparently induced up-regulation of PC3 cell migration and invasion (Figure 7A and 7F), while knock-down of endogenous CXCL13 by siRNA largely weakened PC3 cell migration and invasion (Figure 7F). The effectiveness of CXCL13 transfection and knock-down was confirmed by western-blot assay (Figure 7C and 7E). Quantitative analyses of cell migration and invasion were performed and the quantified data were shown in Figure 7B and 7H. These results indicated that CXCL13 promoted the cell migration and invasion in androgen independent PCa cells. It's reported that re-expression of AR (recovery expression of AR) in androgen independent PC3 cells inhibited cell migration and invasion [33, 34]. Our results here revealed that transient over-expression of AR impaired CXCL13-enhanced cell migration and invasion and finally resulted in obviously inhibition of PC3 cell migration and invasion (Figure 7G), even if transient over-expression of AR still apparently induced up-regulation of CXCL13 in PC3 cells (Figure 3D). The effectiveness of AR transfection and AR induced over-expression of CXCL13 was confirmed by western-blot assay (Figure 7D). Quantitative analyses of cell migration and invasion were shown as Figure 7I. Taken together, these results demonstrated that CXCL13 mediated the enhancement of androgen/AR axis on cell migration and invasion only in androgen dependent PCa cells, rather than in androgen independent PCa cells.

### CXCL13 promoted G2/M phase cell cycle progression and enhanced CWR22Rv1 xenograft tumor growth *in vivo*

**Figure 8:**
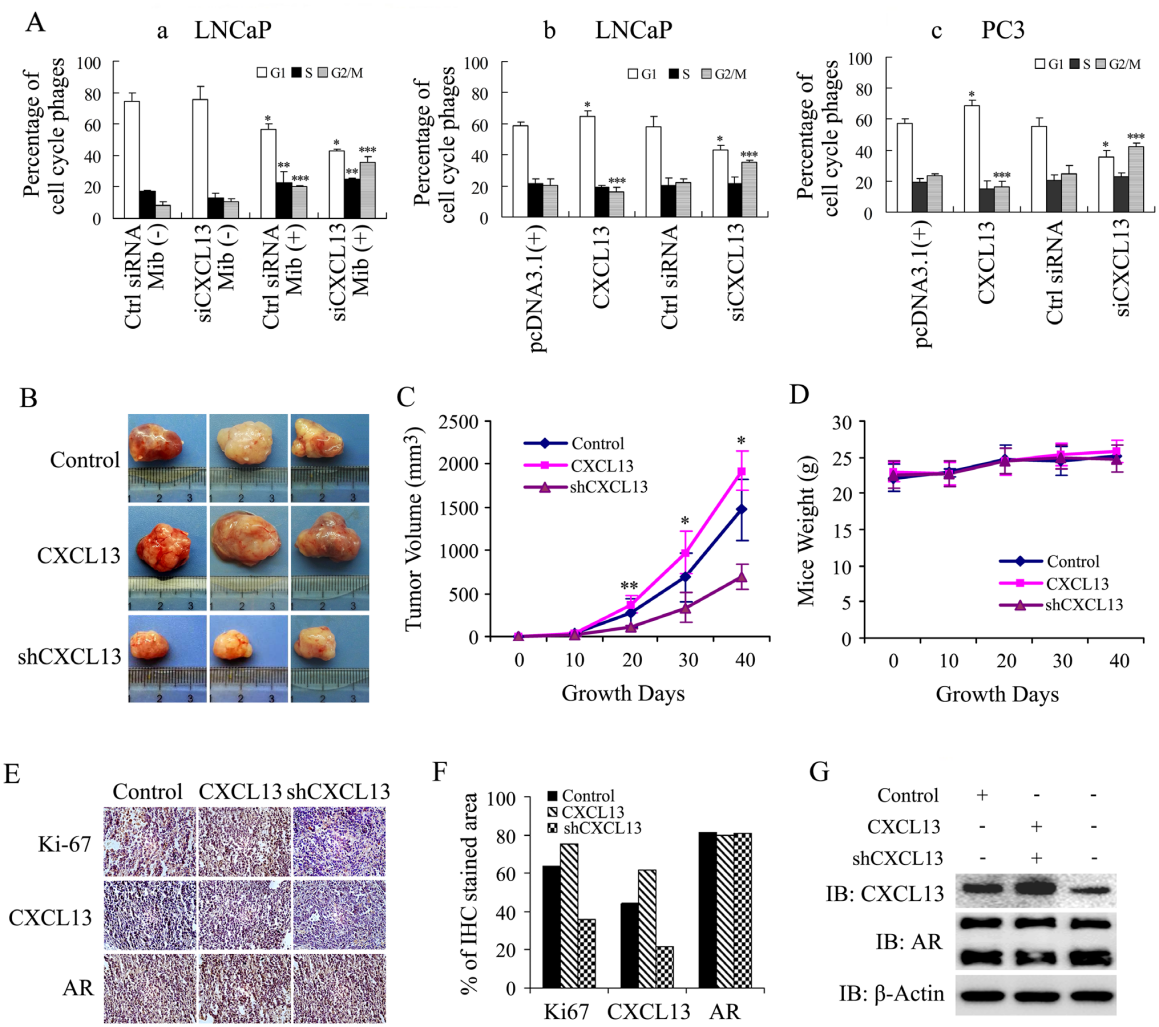
CXCL13 promoted G2 to M phase cell cycle transition and enhanced tumor growth *in vivo* **(A)** LNCaP and PC3 cells were transfected with siCXCL13, control siRNA and plasmids as indicated in figures. **(a)** LNCaP cells were cultured in 10% CSS medium, treated with or without 10 nM of Mib, and then were subjected to flow cytometry analysis. **(b)** LNCaP and **(c)** PC3 cells were cultured in complete medium for 48 hr and then subjected to flow cytometry analysis. Percentages of cells in different cell cycle phases were calculated and presented in figures. **(B-G)** *In vivo* experimental data of the tumor growth xenotransplanted with CXCL13, shCXCL13 and control stable CWR22Rv1 cells. *, **, *** *p* < 0.05. (B) Photograph of tumors after 40 days growth. (C) Growth curve of tumors in 40 days growth. * *p* < 0.05. (D) Mice weight curve in 40 days growth. (E and F) IHC of Ki-67, CXCL13 and AR in tumors. (G) Western blot assay of Ki-67, CXCL13 and AR.

To explore the role of CXCL13 in AR-induced PCa cell cycle transformation, LNCaP cells were cultured in 10% CSS medium immediately after knocking down the endogenous CXCL13 by siRNA. After 48 hr, cells were treated with or without 10 nM Mib for another 24 hr. As show in Figure 8A(a), data of flow cytometry analysis showed that LNCaP cells were mainly distributed in G0/G1 phase in the case of androgen deprivation. After Mib stimulation, G0/G1 phase cells declined while S phase and G2/M phase cells increased significantly, which was consistent with previous reports [35, 36]. Knock-down of endogenous CXCL13 resulted in a further increase of S phase and G2/M phase cells in Mib stimulation cells, whereas the silence of CXCL13 had no significant effect on cell cycle redistribution in androgen-deprivation cells. Moreover, when LNCaP cells were transfected with siCXCL13 or CXCL13 plasmids and cultured in complete medium, the G2/M population was significantly decreased by over-expressed CXCL13 while remarkably increased by the silence of endogenous CXCL13 (Figure 8A(b)); and the similar results were also obtained in PC3 cell (Figure 8A(c)). In addition, Transfection of AR in PC3 cells resulted in a decrease of cells in G1 phase and G2/M phase and a dramatically increase of cells in S phase (data not shown). These data suggest that CXCL13 played a role in promoting G2/M cell cycle transition in all PCa cell lines and also involved in AR-regulated cellular bio-functions via regulating cell cycle transition in androgen dependent PCa cells.

In CWR22Rv1 cell xenograft tumor models of nude mice, over-expression of CXCL13 in CWR22Rv1 cells (stable-expression cells) enhanced tumor growth a little bit and knock-down of CXCL13 by shCXCL13 (shRNA of CXCL13, stable cells) obviously inhibited tumor growth *in vivo* by compared to control group (stable cells with pcDNA3.1(+) and control shRNA), while no changes was observed in the mice body-weight (Figure 8B-8D). IHC assay and western-blot analysis further identified that CXCL13 was over-expressed in CXCL13 stable cells and knocked down in shCXCL13 stable cells. Moreover, over-expression or knock-down of CXCL13 increased or decreased Ki-67 levels respectively, while no effect on AR levels (Figure 8E-8G). Therefore, *in vivo* experimental results also indicated that CXCL13 promoted androgen-dependent PCa cell growth and enhanced androgen-dependent subcutaneous xenograft tumor growth in nude mice.

## DISCUSSION

Chemokines and their corresponding receptors are primary reported to play a pivotal role in chemoattraction and activation of specific leukocytes in various immuno-inflammatory response [37]. Increasing evidences show that they also play an important role in cancer metastasis and tumor cell survival [38, 39]. CXCL13 is a member of CXC chemokine family and is originally named B-cell attracting chemokine 1 (BCA-1). It is expressed by stromal cells within B-cell follicles in secondary lymphoid tissues [40]. The obvious increase of serum CXCL13 in PCa indicates that CXCL13 may be a marker for further diagnostic [26]. Recent studies have shown that CXCL13 not only promotes PCa cell proliferation by activating JNK pathways, but also enhances migration and invasion through ERK signaling [31, 32]. Moreover, studies reveal that CXCL13 up-regulates the expression of MMP-2, MMP-9, and MMP-13 in PCa cell [25]. CXCL13 is tightly correlated with lymph node metastasis which indicates that CXCL13 may contribute to EMT progression [41]. However, there is no reports about the relationship between AR and CXCL13. In this study, we show for the first time that androgen/AR axis can induce the expression of CXCL13 in PCa cells; and CXCL13 is involved in androgen/AR axis to regulate cell growth, cell migration, cell invasion and cell cycle processes in androgen dependent PCa cells. *In vivo* experimental results also show that CXCL13 enhanced PCa cell growth in CWR22Rv1 cell xenotransplanted tumors in nude mice.

CXCL13 is usually expressed in lymphoid organs, such as lymph nodes and spleen, especially by stromal cells and follicular DC (FDC) in B cell follicles [42]. Further, it has reported that CXCL13 is usually highly expressed in human bone marrow endothelial (HBME) cells and osteoblasts (OBs) and then secreted into serum resulting in a higher serum CXCL13 level in PCa patients [26]. Our results here show that CXCL13 can be expressed by PCa cells themselves, especially in androgen dependent PCa cells. From the clinic samples to cancer cell lines, CXCL13 is higher expressed in PCa tissues and PCa cells than that in Pericarcinomatous tissues and normal prostate cells (WPMY-1 cells). And importantly, CXCL13 levels in androgen-responsive PCa cell lines is much higher than those in androgen-unresponsive PCa cell lines (Figure 1). Furthermore, when using the medium (cultured CXCL13-transfected PC3 cells) to culture non-transfected PC3 cells, we find that the migration and invasion of non-transfected PC3 cells are much more enhanced than that of normal medium cultured PC3 cells (data not shown). All these are telling us that the expression of CXCL13 is high correlated to androgen and AR, and also implying that CXCL13 may be relevant to the androgen/AR axis-regulated PCa occurrence and development in clinic. Additionally, the difference of CXCL13 expression among androgen-dependent PCa cells, androgen-independent PCa cells and normal prostate cells (Figure 1D and 1E) indicates that CXCL13 expression is not only regulated by androgen/AR axis, but may be also regulated by other different transcription factors via androgen-independent signal pathways.

As we know, AR plays an important role in cell migration and invasion in androgen-dependent PCa cells [2, 3]. The increasing expression of CXCL13 observed in several human tumors (including breast cancer, colon cancer and PCa) has been implicated in cancer progression by inducing cell migration and invasion [26, 43]. Our experiment data show that the androgenic effect (activated androgen/AR axis) on cell migration and invasion is weakened by decreasing endogenous CXCL13 in androgen-dependent PCa cells (LNCaP cells); and over-expressing CXCL13 alone promotes cell migration and invasion in androgen-independent PCa cells (PC3 cells). However, transient over-expression of AR in PC3 cells exhibits a totally inhibition of cell migration and invasion although high expression of CXCL13 is still induced by transfected AR (Figure 7). These results indicate that CXCL13 is involved and cooperated with AR in androgen/AR axis-induced enhancement of cell migration and invasion in androgen-dependent LNCaP cells; whereas the enhancement of cell migration and invasion by AR-induced CXCL13 in androgen-independent PC3 cells is shadowed by AR-inhibited cell migration and invasion. AR-mediated inhibition of cell migration and invasion plays a leading role when CXCL13 and AR levels are simultaneously increased in androgen-independent PCa cells (PC3 cells). It may suggest that androgen/AR axis initiates different molecular mechanism and signal pathways to regulate the cell migration and invasion in LNCaP and PC3 cells, respectively.

As we know, cyclin B1 is related to cell cycle and cell phase transition (G2/M) in cell proliferation [44, 45], while Ets-1 and Snail are related to cell growth, proliferation, epithelial-mesenchymal transition (EMT), and cancer cell migration and invasion [46, 47]. From our results, transient over-expression of AR or CXCL13 increases the expression of cyclin B1, Ets-1 and Snail in either androgen-dependent LNCaP cells or androgen-independent PC3 cells. Knock-down of CXCL13 by its siRNA weakens the androgen/AR axis-enhanced expression of cyclin B1, Ets-1 and Snail in LNCaP and CWR22Rv1 cells (Figure 5). These results indicate that CXCL13 is involved not only in cell cycle and cell phase transition, but also in cancer cell growth, proliferation, EMT, migration and invasion. As we know, it's necessary for CXCL13 to carry out its activity and bio-functions that CXCL13 binds with its receptor CXCR5 [20, 23, 24]. So, the molecular mechanism for CXCL13 binding CXCR5 to regulate the expression of *cyclin B1*, *Ets-1* and *Snail* genes needs to be further explored in the next study.

In summary, our study demonstrated that CXCL13 is a downstream target gene of androgen/AR axis, and the expression of CXCL13 is up-regulated by androgen/AR axis in both androgen-dependent and -independent PCa cells. CXCL13 plays an important role and involves in androgen/AR axis-induced cell cycle, cell migration and invasion in androgen-dependent PCa cells. However, in androgen-independent PCa cells, inhibition of cell migration and invasion by recovering AR is the leading factor as opposed to CXCL13-enhanced cell migration and invasion when CXCL13 and AR are co-expressed in androgen-independent PCa cells. Moreover, different expression of CXCL13 between PCa tissues/cells and normal prostate tissues/cells implies that CXCL13 plays a role and involves in the carcinogenesis of prostate and the progression of PCa. Further understanding the detailed roles of CXCL13 in carcinogenesis of prostate and PCa development may lead to the identification of a novel therapeutic target for PCa treatment as well as a potential prognostic marker.

## MATERIALS AND METHODS

### Cell culture, transfection and lentivirus infection

The human PCa cell lines, LNCaP, CWR22Rv1, PC3, DU145, and normal prostate epithelial cell line WPMY-1, were obtained from American Type Culture Collection (Manassas, VA, USA). LNCaP and CWR22Rv1 cells were maintained in RPMI-1640 medium supplemented with 10% fetal bovine serum (FBS), 100 mg/ml streptomycin, and 100U/ml penicillin. PC3, DU145 and WPMY-1 cells were grown in Dulbecco's Modified Eagle's Medium (DMEM; Invitrogen Inc., Carlsbad, CA, USA) containing 10% FBS, penicillin (100U/ml) and streptomycin (100 mg/ml). All cell lines were cultured in a humidified 5% CO_2_ air atmosphere at 37°C. For androgen dose and time-course studies, cells were plated in medium with 10% charcoal-stripped serum (CSS; androgen deprived from the serum); after 48 hr, mibolerone (Mib, a potent synthetic androgen, dissolved in ethanol) or ethanol (EtOH) was added.

Cell transient transfection were performed by using Lipofectamine 2000 (Invitrogen Inc., CA). Small interfering RNA (siRNA) specific for AR, CXCL13 and control siRNA were purchased from Santa Cruz Biotechnology Inc. (Santa Cruz, CA). Approximately 70–90% transfection efficiencies were achieved.

Lentiviruses carrying shRNA targeting human CXCL13 lentiviral vectors (GV112) were from Genechem Co., Ltd. (Shanghai, China). The viruses were used to infect cells in the presence of Polybrene. After 48 hr, CWR22Rv1 cells were cultured in medium containing puromycin for the selection of stable clones.

### Plasmids, antibody, siRNAs and chemicals

Expression plasmids, including pcDNA3.1(+)-CXCL13, pcDNA3.1(+)-AR, pcDNA3.1(+)-AR-N, pcDNA3.1(+)-FOXO1 and pcDNA3.1(+)-HDAC1, were constructed by and stored in our lab. Series of dual-luciferase test plasmids of AR binding elements in CXCL13 enhancer (including wild-type and mutants) were constructed in pGL3-promoter vector (purchased from Promega, WI, USA). For CXCL13 stable-expression cell line, the plasmid pcDNA3.1(+)/CXCL13 was transfected into CWR22Rv1 cells. After 48 hr, cells were cultured in medium containing G418 for the selection of stable clones.

Antibodies against AR (N-20), AR(441), FOXO1, HDAC1, β-actin, ETS-1 and Cyclin B1 were purchased from Santa Cruz Biotechnology Inc. (Santa Cruz, CA, USA). Snail was assessed with an antibody from Cell Signaling Technology, and CXCL13 with an antibody from Sigma-Aldrich (Sigma-Aldrich, Munich, Germany). The appropriate secondary antibodies were all purchased from Santa Cruz Biotechnology Inc. (Santa Cruz, CA). All siRNAs (including CXCL13 specific siRNA, AR specific siRNA and control siRNA) were purchased from Santa Cruz Biotechnology Inc. Mibolerone (Mib) was purchased from Steraloids Inc. (Newport, RI, USA). Other chemicals were all purchased from Sigma-Aldrich. The molecular weight (protein size) of same proteins was labeled when it first appeared in figures.

### Transcriptome sequencing, immunohistochemistry and western-blot analysis

The study on clinical PCa specimens and experimental animals was approved (Permission No: NL-129-02) by the Ethics Committee of Jiangsu Province Hospital of TCM, Nanjing, China, and written informed consent were obtained from the patients. The primary PCa specimens, which were excised from patients and confirmed including different Gleason Score from 7 to 10, were selected as PCa group and the normal tissue next to the tumor (more than 5 cm from the edge of the tumor). 137 specimens including PCa and matched adjacent normal tissues were carried out for real-time RT-PCR assay. Two groups of specimens (5-PCa group and 5-adjacent normal tissue group) were carried out for transcriptome sequencing assay by KangChen Bio-tech Inc., Shanghai, China. Three PCa samples and three adjacent normal samples were carried out for immunohistochemistry (IHC) assay with anti-CXCL13 antibody (1:100 dilution) and routine hematoxylin staining.

For western blot assay, cell was harvested and lysed in lysis buffer. After centrifuge, the total protein of supernatants were quantified using DC protein assay kit and the supernatants (containing total protein 30-60 μg) were treated for SDS-PAGE (β-actin as the internal control) and transferred to nitrocellsulose membranes. Membranes were then blocked with skimmed milk, incubated with primary antibody (1:1000 dilution) overnight, washed with TBST for three times 5 min each time, incubated with 2nd antibody for 1 hr, and washed with TBST for three times 7 min each time. Luminescence were developed with Enhanced Chemiluminescence Detection Kit (Amersham Bioscience, Pittsburgh, PA, USA).

### Semi-quantitative and real-time RT-PCR

Total RNA was isolated with Trizol reagent (Invitrogen, CA). cDNA was synthesized using SuperScript II reverse transcriptase (Invitrogen). RT-PCRs were performed as described by the manufacturer. The following primer sequences were used: AR, forward 5′-CGG ACG AGG ATG ACT CAG-3′ and reverse 5′-TCT TCA GTG CTC TTG CCT GC-3′; CXCL13, forward 5′-ATG AAG TTC ATC TCG ACA TCT CTG CTT CTC-3′ and reverse 5′-AGG GAA TCT TTC TCT TAA ACA CTG GAA CTG G-3′; β-actin, forward 5′-GAG CTA CGA GCT GCC TGA CG-3′ and reverse 5′-CCT AGA AGC ATT TGC GGT GG-3′.

### Luciferase assay

LNCaP and PC3 cell were plated on 12-well plates and transfected using Lipofectamine 2000 (3 mL per well; Invitrogen). The total amount of plasmid DNA was normalized to 1.5 μg per well. After 48 hr, cell were lysed in lysis buffer provided in the Luciferase Assay System (Beyotime Biotechnology). Luciferase activities were measured using the Dual-luciferase Reporter Assay System (Beyotime Biotechnology) with the aid of a microplate luminometer (Tecan, Switzerland), and Renilla luciferase activities of cell were used as internal control. All experiments were carried out in triplicate wells and repeated 3 times.

### ChIP-seq and ChIP assay

LNCaP cells were treated with 10 nM Mib or ETOH for 3 hr after 48 hr of plating in 10% CSS medium and then harvested for CHIP-seq experiment. We performed AR, H3K4me1, H3K4me3 and H3K27me3 ChIP-seq by using an Illumina Genome Analyzer or Hiseq (Illumina, SanDiego, CA). According to the Illumina's instructions, we prepared sequence data Libraries. ChIP-Seq sequencing reads were mapped to the human genome (GRCh37/hg19) using Bowtie2 with default parameters. Peak finding and annotation to the nearest RefSeq gene promoter were carried out using HOMER. Motif analysis of binding sequences was also performed using HOMER. More details were described as in references [48].

For ChIP assay, LNCaP cells were treated with 10 nM Mib or ETOH for 3 hr after 48 hr of plating in 10% CSS medium and the ChIP assay was performed as described previously [49]. Briefly, cells were sonicated and the soluble chromatin was incubated with 2 μg of rabbit IgG or AR (441) antibodies. PCR was performed using primers specific for the AR binding region 1 in the CXCL13 enhancer: forward 5′-AGC CGT GGT ATC AGG ACG TT-3′; reverse 5′-TTG CTG TCT TAG CCA TCC CA-3′. Real-time PCR was performed with ChIP samples.

### Cell proliferation assay

Cell proliferation was measured by MTT assay. At the indicated time, the medium in each well was removed and replaced with 0.5 mL of fresh phenol red–free medium containing 0.5 mg/mL MTT, and then cells were incubated at 37°C for 5 hr. The medium was discarded, and then 0.2 mL DMSO was added to each well to dissolve the formazan dye trapped in the living cells. Then 100 μL of the supernatants was transferred into a 96-well plate and read at A570.

### Cellular migration by wound healing assay

LNCaP cells were plated and cultured in medium with 10% charcoal-stripped serum (CSS). After transfection with control siRNA or CXCL13-specific siRNA (siCXCL13) for 48 hr, cell were cultured in a 6-well plate until confluent. The cell layer was carefully wounded using a sterile tip and washed twice with fresh serum free media; and then cultured in medium with 10% CSS and treated with vehicle (EtOH) or 10 nM Mib. Photographs of the same area of the wound were taken at 0 day, 3 days, and 5 days after wounding to measure the width of the wound. The areas of the gaps and percent wound areas filled were determined.

### Cellular migration and invasion by transwell assay

Cell migration and invasion assays were performed using transwell chambers (pore size of 8 μm; Costar, Corning, Switzerland), which bottom was coated with 1 mg/ml BD Matrigel Matrix (BD Biosciences, USA) for invasion assay specifically. The inserts were placed in the 24-well culture plates. LNCaP cells were transfected with control siRNA or siCXCL13. After 48 hr, cells were trypsinized and re-suspended in phenol red–free and serum free RPMI 1640 medium. 200 μl of the cell suspension (containing the same number of cells) was added to the upper chamber with uncoated (for migration assays) or Matrigel coated (for invasion assays) membranes. Meanwhile, 500 μl of phenol red–free medium containing 20% CSS with or without 10 nM Mib was added to the lower chamber as chemoattractant. After incubating 24 hr, cells that had not migrated through the pores were manually removed from the upper face of the filters using cotton swabs, and cells adherent to the lower surface of the filters were fixed in cold 100% methanol for 30 min and then stained with 0.1% crystal violet for 30 min. Finally the filters were washed thoroughly in 1×PBS and images were taken under a microscope with digital imaging system (Olympus DP50, Olympus, Japan) in the appropriate magnification. Then the inserts were immersed in 33% acetic acid for decolorization treatment until the crystal violet was completely eluted, the eluent was then measured at 570 nm on a microplate reader, and the OD values indirectly reflects the number of migrated and invaded cells. These experiments were done in triplicate and performed a minimum of three times.

### Cell cycle assay and *in vivo* experiments

LNCaP (two groups) and PC3 cells were transfected with siRNAs and plasmids as indicated in Figure 8A, and then cultured in the medium containing 10% CSS (one group of LNCaP cells) or 10% FBS (one group of LNCaP cells and PC3 cells), respectively. After 48 hr, LNCaP cells cultured in 10% CSS medium were treated with or without 10nM Mib for additional 24 hr. Culturing 48 hr for cells with 10% FBS medium and 72 hr for cells with 10% CSS medium after transfection, cells were harvested and fixed in 70% ethanol overnight, then treated with RNase (10 mg/mL). Propidium iodide (250 g/mL) was then added to the samples, and the samples were mixed and incubated in the dark for 30 minutes. Flow cytometry analysis was performed using the EPICS Elite ESP high-performance cell sorter (Coulter Electronics Ltd., England, UK). The raw collected data were analyzed by ModFit LT (version 2.0; Verity Software) to eliminate aggregated cell for determination of cell cycle distribution.

For *in vivo* experiments, CWR22Rv1 cells were transfected with pcDNA3.1 (+)/CXCL13 and lentivirus vectors with shCXCL13 gene as indicated in Figure 8B-8G (control was the cells stable transfected with both pcDNA 3.1(+) and control shRNA). CXCL13 stable over-expression and knock-down cell lines were got from positive clones of CXCL13 level assayed by western blot. The control cells were obtained from positive clones of double selection with G418 and puromycin (transfected with pcDNA3.1(+) and infected with lentiviruses carrying control shRNA). And then, the stable expression cells were cultured and finally 1×10^6^ live cells in 100 mL 1×BSS were injected subcutaneously into 6-weeks-old nude mice (supplied by the animal center in the College of Medicine, Nanjing University, Nanjing, China). The tumor size and mice weight were measured at 0, 10, 20, 30 and 40 days. After tumor growth for 40 days, the mice were sacrificed and subcutaneous tumors were isolated and photographed; and then the tumors were equally dissected into two parts. One part of the tumor tissues were frozen in liquid nitrogen and stored at −80°C for western-blot assay, and another part were formalin fixed and paraffin embedded for immunohistochemistry (IHC).

### Statistical analysis

All data were analyzed using Stata 7.0 statistics software. Values were expressed as means SD. Comparison between two mean values was made by independent-sample *t*-test. A *p* value of <0.05 was considered to be statistically significant.
